# Drug addiction in Gaza and the illicit trafficking of tramadol

**Published:** 2010

**Authors:** Yusef Progler

**Affiliations:** a Professor of Comparative Cultures at Asia Pacific University in Japan and Co-creator of Multiversity in Malaysia and India. E-mail: yjprogler@gmail.com

Following the December 2008 Israeli offensive, a United Nations survey of Gaza residents found increases in risk taking behaviour, including a significant rise in cases of drug addiction.^1^ One drug associated with this trend is Tramadol, first developed in Germany during the 1970s and introduced in the 1990s as a centrally acting analgesic with properties similar to codeine and morphine and which is widely prescribed as a pain killer.^2^ Although illegal without a prescription in some regions, Tramadol is relatively easy to obtain in Gaza, either with fake prescriptions from pharmacies or on the black market. News reports prior to the 2008 offensive suggested that up to 30 percent of males between the ages of 14 and 30 had already been using Tramadol on a regular basis, with some 15,000 showing signs of addiction.^3^ The escalation of this problem since then was documented by the Aljazeera English satellite television channel in its recently aired program “Uncomfortably Numb.”

Under the broad ranging and comprehensive Israeli blockade of the Gaza strip, which has been in place since 2007, Gazan society has come to rely heavily on a vast network of cross border tunnels, some as much as 18 meters underground, which have become crucial supply routes for Palestinians who are cut off from the outside world. Around the clock shifts of tunnel workers transport a wide variety of items, from food and clothing to household goods and even furniture. Recently, illicit pharmaceuticals and other drugs have become hot commodities in the burgeoning tunnel trade.

Despite consistent border seizures of the smuggled drugs, thousands of boxes of Tramadol still make their way to Gazans who are increasingly dependent on the drug, some having become seriously addicted. Kamal, an addict interviewed in the program, admitted that, “When I take it, I feel completely relaxed. I forget about all my feelings and emotions. I feel total release when I take one or two pills. The higher the dosage the better I feel, the more relaxed.” Dr Samir Al Zaqout of the Community Health Programme in Gaza, one of the few facilities at which addicts can receive treatment, says of the rising tide of addiction, “Most of the addicts are between the ages of 18 and 30. All my cases are in that age bracket, and herein lies the danger. The danger is that those who are supposed to build our future are the most affected. And if the number of cases I have seen are 150, there are hundreds of others who I have not seen and who would never seek the help of a doctor. Why? Because we live in a traditional society that fears the stigma attached to mental illness, and addiction is not just considered to be a mental health issue. It’s seen as even more serious.” Kamal continues, “I take it because of the hardship we’re under. I take it because of what is happening to us. I used to take one or two a day; now I need six. Since the war we have been in a miserable situation. No jobs, Hamas and Fatah fighting, no reconciliation, nothing. We were promised that things would open up and more jobs would become available for me and others. We want the wall to be opened, we don’t want the siege.” Dr Zaqout adds, “The number of addicts went up after the war in Gaza because that war was unprecedented. The occupation employed every form of discriminate and indiscriminate killing leaving the Palestinians here feeling insecure and at risk wherever they are.”

While it was not mentioned in the program, it is worth noting here that researchers have found a very high degree of comorbidity between war-related trauma and depression, along with drug abuse, among Israeli soldiers who, ironically, have also been psychologically affected by the ongoing war.^4^

However, as the Aljazeera reporter Zeina Awad notes, “It’s not just the war. Israel’s suffocating siege has cut Gazans off from the outside world and made hundreds of thousands of them jobless. Gazans are becoming poorer by the day, and eight out of ten Gazans are now dependent upon some form of UN handouts.” University graduates have increasingly difficult times finding jobs, and end up hanging around in cafés. One student interviewed in the program recalls that over 3000 university graduates showed up for a recent job fair at which there were only 100 positions available. Some students turn to drugs to ease their frustration. As suggested by a second student, “Even those who don’t do bad things are now thinking about doing them. The war has deeply affected us. Our spirit is destroyed. We have not left since the war, nor had a break. And we’re still carrying it all inside us.” Another student reports that her brother brought pills home from school that turned out to be Tramadol, which led to an investigation at the school that found a 15 year old student who was dealing in drugs.

The market for Tramadol was at first driven by user need, but when demand outstripped supply a black market arose with an interest in proliferation. This fuelled a lucrative criminal economy of illicit drugs, with dealers getting rich on users who may spend what little cash they have on drugs rather than daily essential needs such as food and clothing. While addicts live one day at a time, many not knowing from where their next fix will come, a battle has emerged between Hamas police forces and the increasingly wealthy and powerful drug dealers. Hamas has taken a hard line on the illegal drug trade, including shooting dead on site known large scale dealers and using torture on drug suspects. Hamas is also attempting to change the drug laws in Gaza, from the looser Israeli norms to those from Egypt that permit life jail sentences for drug dealers and even the death penalty in some cases.

Although the methods employed by Hamas have raised concerns, its crackdown has succeeded in bringing a semblance of order to the formerly lawless streets of Gaza and has reduced the drug supply by 80%, according to a spokesman for the police, who also denied torture was being used. When Fatah was in control of Gaza, drug addicts were often labelled as “collaborators” with Israel, creating a double edged – and potentially deadly – social stigma. The association between addiction and collaboration was continued by Hamas. But such policies have adverse effects, as noted by Dr Zaqout: “Psychologically, torture would only increase the person’s addiction, so you are making matters more complicated by beating him. If the addict does not feel that society sees him as a human being, then he or she will become more depressed.” Coupled with the hardships of the ongoing Israeli occupation, suffering abuse by the Palestinian police and living with social stigma can have the effect of aggravating the problems people face, leading to deeper depression and addiction.

Whatever the tactics and results, the demand for drugs continues and as with any illicit drug trade anywhere in the world, as long as there is demand there will be dealers willing to take whatever risks are necessary to meet that demand and reap its immense profits. In one sequence of the program, Zeina Awad is taken along on a drug bust operation that does not yield any evidence of dealing, but she is later shown what has been seized from previous raids, including supplies of “ecstasy” pills and large quantities of hashish, and countless boxes of Tramadol. Unfortunately, as noted by the Head of the Anti-Drug Task Force, Jamil Al Dahshan, “There is a big difference between the number of arrests in 2009 compared to 2008. Drug cases have gone up in the last year. In 2009, we had close to 1,204 cases of which 591 were Tramadol related. We seized close to two and half million Tramadol pills compared to 550,000 in 2008. We had 734 drug cases in total. 109 of those were Tramadol related.”

While the program was informative, Al-jazeera’s singular focus on the tunnels as the main entry point for Tramadol and other substances into Gaza missed an important opportunity to explore drug trafficking as a transnational crime,^5^ evidence of which was clearly shown in the program. Although Tramadol is known as Tramal in Gaza and Tramadex in Israel, the police displayed boxes labelled Tramajack, which is the name under which Tramadol is manufactured in India. The police also showed packets of green capsules labelled Tramadol, but which appear to have originated in the European Union. Other drugs on display were Proxam, a compound including dextropropoxyphene hydrochloride, which is a weak opioid, and Amirol, which is the Australian name for amitriptyline hydrochloride, a powerful antidepressant with sedative properties. In addition to drugs, the police confiscated substances related to the drug trade, such as Marquis Reagent, which is used for testing ecstasy, cocaine and opiates, and even reagents for testing the presence of explosives. However, Al Dahshan suggests that, “The drugs are mostly supplied by Israel, indirectly via Egypt, and the tunnels. Some drugs also come directly from Egypt. The profits tempt young people, who want to get rich quick. We have caught dealers between the ages ranged from 20-23 with huge quantities of drugs and they are considered among the biggest dealers in Gaza. The Israelis are the main source of drugs to Gaza and their aim, as our evidence from official cases shows, is to flood Gaza with drugs.”

Beyond meeting with addicts, doctors and police, Aljazeera interviewed workers in the tunnels who admitted taking Tramadol to help them make it through their all night jobs. Awad also visited the Gaza central jail, in a section where 120 prisoners (users and dealers) are held on drug charges. One prisoner says he had been taking drugs for over 10 years, and first took them while working in Israel. Khalid, another addict interviewed, concludes by saying, “I would like to be able to stop taking drugs, because I am tired. I am depleted from the inside. I’m talking to you and all of my internal organs are at God’s mercy. Sometimes I feel that my wife will try to wake me up but I won’t wake up. I will be gone and meet God Almighty.” Kamal, who despite the problem of social stigma is seeking regular treatment to overcome his addiction, puts a human face on the whole affair, and says that the hope lies in improving life in Gaza: “The first thing I wish for is a job. The second dream I have is to settle in a house of my own, that I can have a nice home to live in and to leave the Tramadol pills behind.”

“Uncomfortably Numb” first aired on the Aljazeera English satellite television channel in January 2010 and is currently available on the Aljazeera English website^6^ and viral media sites such as YouTube.^7^
